# Stellenwert der Elektrokonvulsionstherapie (EKT) in der forensischen Psychiatrie

**DOI:** 10.1007/s00115-020-00947-4

**Published:** 2020-07-08

**Authors:** Matthias Besse, Anna-K. Schomburg, Alfred Simon, Dirk Hesse, Jürgen Müller, David Zilles-Wegner

**Affiliations:** 1 Klinik für Psychiatrie und Psychotherapie, Universitätsmedizin Göttingen, Von-Siebold-Straße 5, 37075 Göttingen, Deutschland; 2 Institut für Ethik und Geschichte in der Medizin, Universitätsmedizin Göttingen, Humboldtallee 36, 37073 Göttingen, Deutschland; 3 Akademie für Ethik in der Medizin e. V., Humboldtallee 36, 37073 Göttingen, Deutschland; 4 Maßregelvollzugszentrum Niedersachsen – Moringen, Mannenstraße 29, 37186 Moringen, Deutschland; 5 Asklepios Klinik für forensische Psychiatrie und Psychotherapie, Rosdorfer Weg 70, 37081 Göttingen, Deutschland

**Keywords:** Elektrokrampftherapie, Therapieresistenz, Maßregelvollzug, Fragebogen, Leitlinien, ECT treatment, Therapy resistance, Hospital treatment order, Questionnaire, Therapy guidelines

## Abstract

**Hintergrund und Ziel der Arbeit:**

Für die Elektrokonvulsionstherapie (EKT) liegen Leitlinienempfehlungen zur Behandlung schwerer affektiver und psychotischer Erkrankungen vor, wie sie auch bei Patienten im Maßregelvollzug (MRV) vorkommen. Über die Anwendung der EKT in der forensischen Psychiatrie existieren jedoch keinerlei systematisch erhobene Daten. Ziel der vorliegenden Arbeit ist eine Erhebung des Ist-Zustands sowie des geschätzten Bedarfs an EKT im MRV.

**Material und Methoden:**

Es wurde ein Fragebogen zur Erhebung der aktuellen Anwendung sowie der Einschätzung des Bedarfs an EKT in dieser Population entwickelt. Dieser wurde elektronisch und bei Bedarf postalisch an die forensischen Kliniken in Deutschland versendet.

**Ergebnisse:**

Wir erhielten Antwort von 52 Kliniken (66 %). 29 der Kliniken gaben an, Patienten mit EKT behandeln zu können. Eine Behandlungsindikation wurde klinikübergreifend für 3,4 % der untergebrachten Patienten gesehen. In einem Jahr (2018) wurden insgesamt 32 EKT-Behandlungen an 12 Kliniken des Maßregelvollzugs mit durchschnittlich guter Wirksamkeit (Ø CGI‑I 2,32) durchgeführt. Häufigste Begründung für die fehlende Anwendung der EKT waren mangelnde strukturelle Voraussetzungen. Jeweils etwa ein Drittel der Kliniken sehen einen mittleren bzw. hohen Bedarf an EKT im MRV.

**Diskussion:**

Die EKT kommt im MRV in Deutschland aktuell nur sehr begrenzt zum Einsatz. Auffällig ist, dass die Anzahl der durchgeführten Behandlungen etwa um den Faktor 8 hinter der Indikationshäufigkeit zurücksteht. Ungeachtet dessen sehen zwei Drittel der Kliniken einen mittleren bis hohen Bedarf, womit eine Unterversorgung mit EKT im MRV zu bestehen scheint.

## Abstract (en)

**Background and objective:**

Electroconvulsive therapy (ECT) is recommended in guidelines for the treatment of severe affective and psychotic disorders that also can be found in patients in forensic psychiatric commitment; however, there are no systematically collected data concerning the use of ECT in forensic psychiatry. The aim of this study was to investigate the current state and estimated need for ECT in forensic psychiatry in Germany.

**Material and methods:**

A questionnaire was designed consisting of questions about the current practice and estimated need for ECT in this population. This questionnaire was sent electronically (and by post if needed) to the forensic psychiatric hospitals in Germany.

**Results:**

We received replies from 52 hospitals (66%) and 29 of these hospitals stated that they were capable of treating patients with ECT. An indication for ECT treatment was seen in 3.4% of all detained patients across all forensic hospitals. In 1 year (2018) 32 ECT treatment courses were carried out with on average good effectiveness and a mean clinical global impressions (CGI) scale of 2.32. The most frequent reason for not using ECT was lack of structural requirements. Approximately one third of the forensic psychiatric hospitals stated that there was a medium or even high need for ECT in forensic psychiatry.

**Conclusion:**

The use of ECT in German forensic psychiatry is currently very limited. It is striking that the number of applied ECT treatments is eight times lower in comparison to the frequency of indications. Nevertheless, approximately two thirds of the forensic hospitals stated a medium or high need for ECT, which seems to suggest a shortage of ECT in the treatment of forensic psychiatry patients.

## Einleitung

Die Behandlung im Maßregelvollzug (MRV) erfolgt auf Grundlage des § 63 Strafgesetzbuch bei Patienten, die im Zustand der vollständigen oder erheblich verminderten Schuldfähigkeit aufgrund einer überdauernden psychischen Störung eine erhebliche Straftat begangen haben und störungsbedingt weiter gefährlich sind. In Deutschland betraf dies im Jahr 2017 etwa 6300 Patienten nach § 63 StGB.

Darüber hinaus waren etwa 3900 Patienten in einer Entziehungsanstalt nach § 64 StGB untergebracht [18]. Gemäß § 64 wurde bei diesen Patienten ein Hang zur Einnahme berauschender Mittel festgestellt und sie hatten eine Straftat begangen, die auf den Hang zurückging. Außerdem waren weitere mit dem Hang bedingte Straftaten zu erwarten und es bestand eine Aussicht auf Behandlungserfolg.

Basierend auf dem Kerndatensatz, in den zwar nicht alle, aber die große Mehrzahl der Bundesländer einbezogen sind, lässt sich für die letzten Jahre ein steigender Anteil untergebrachter Patienten mit der Diagnose Schizophrenie feststellen. Bezogen auf die absolute Zahl kam es zu einem Anstieg von etwa 23 % zwischen 2006 (2299 von insgesamt 7304 Patienten) und 2016 (2817 von 5392 Patienten; [8]).

Die Unterbringung und Behandlung im MRV ist in den entsprechenden Ländergesetzen geregelt. Gemäß § 8 Nds. MVollzG etwa haben im MRV untergebrachte Personen Anspruch auf die nach dem aktuellen Stand des Wissens notwendige medizinische Behandlung ihrer psychischen Störung, derentwegen die Unterbringung notwendig ist (Anlasskrankheit). Behandlungen und Untersuchungen bedürfen der Einwilligung der untergebrachten Person, bei Einwilligungsunfähigkeit der Einwilligung des rechtlichen Vertreters. Unter definierten Voraussetzungen kann auch eine Behandlung gegen den natürlichen Patientenwillen erfolgen (§ 8 a Nds. MVollzG). Ziel der Behandlung ist es u. a., die untergebrachte Person entlassungsfähig zu machen und die Dauer der Freiheitsentziehung auf das unbedingt erforderliche Maß zu begrenzen. Ein individueller Behandlungsplan soll zügig und konsequent umgesetzt werden; Aufwand und Kosten dürfen keine Rolle spielen, sofern über Standardtherapien hinaus individuelle Therapieangebote entwickelt oder angewandt werden sollen [19].

Die Elektrokonvulsionstherapie (EKT) ist ein etabliertes Therapieverfahren zur Behandlung schwerer und pharmakotherapieresistenter depressiver und psychotischer Störungen. Die aktuelle S3-Leitlinie Schizophrenie der DGPPN [10] empfiehlt die EKT zur Augmentierung bei medikamentöser Behandlungsresistenz mit dem Ziel der Verbesserung des klinischen Gesamtzustands. Wurde in der Leitlinie von 2006 [9] die EKT noch primär als Therapie der Wahl bei (perniziöser) Katatonie genannt, hat sich aufgrund neuer und methodisch hochwertigerer Studien die Evidenzbasis deutlich verbessert [14, 20], was in geringerem Ausmaß auch für die Erhaltungsbehandlung gilt [4, 23]. Insofern hat die EKT in den letzten Jahren wieder einen deutlich höheren Stellenwert in der Behandlung schizophrener Störungen gewonnen.

Die Datenlage zur Anwendung der EKT in der forensischen Psychiatrie ist national wie international sehr limitiert. In einem Editorial des *Journal of ECT* äußert der Autor für die USA die Vermutung, das Thema sei nicht nur vernachlässigt, sondern vielmehr aktiv gemieden worden, da es unbequeme Fragen ohne einfache Antworten mit sich bringe [16]. Hinsichtlich der EKT im MRV in Deutschland gibt es unserer Kenntnis nach nur eine einzige Publikation, in der ein vielfach fehlender Zugang zur EKT in der forensischen Psychiatrie konstatiert wird. Eine Indikation zur EKT wurde in der untersuchten forensischen Klinik bei 3,2 % der untergebrachten Patienten insgesamt und bei 11,5 % der Patienten mit Schizophrenie oder affektiver Störung gesehen [25].

Vor dem Hintergrund eines mutmaßlich noch immer häufig fehlenden Zugangs zur EKT, des steigenden Anteils von Patienten mit (pharmakotherapieresistenter) Schizophrenie im MRV sowie der neuen Studien und Leitlinienempfehlungen zur EKT aus dem Bereich der Allgemeinpsychiatrie stellen wir die Hypothese auf, dass die EKT im MRV unterrepräsentiert ist, d. h., relativ zur Indikationshäufigkeit nur selten zur Anwendung kommt. Ziel der vorliegenden Studie ist daher eine erste systematische empirische Datenerhebung zum aktuellen Einsatz der EKT und dem Bedarf im MRV in Deutschland sowie der Identifikation möglicher Hindernisse in der Etablierung.

## Material und Methoden

Zum Zweck der Datenerhebung wurde von unserer Arbeitsgruppe ein Fragebogen erstellt. Der Fragebogen wurde im Januar 2019 zunächst per E‑Mail an die Leitungen von 79 Kliniken des Maßregelvollzugs in Deutschland geschickt. Als Grundlage für die Klinikauswahl diente der E‑Mail-Verteiler des Arbeitskreises Forensische Psychiatrie, in dem alle forensischen Kliniken in Deutschland vertreten sind. Eine erste Erinnerung zur Studienteilnahme erfolgte per E‑Mail im Februar 2019. Zusätzlich wurden auf postalischem Wege im März 2019 diejenigen Kliniken kontaktiert, von denen bis zu diesem Zeitpunkt kein Rücklauf erfolgte.

Der Fragebogen enthielt insgesamt acht Fragen mit folgendem Inhalt:

- Einschätzung der Wirksamkeit der EKT bei Depression und schizophrenen Störungen auf einer visuellen Analogskala (0 = keine Wirksamkeit, 10 = sehr gute Wirksamkeit)
- Vorerfahrungen der Studienteilnehmer mit EKT
- Geschätzter prozentualer Anteil der Patienten in der jeweiligen Klinik, für die eine EKT-Indikation besteht
- Möglichkeit zur Durchführung einer EKT an der jeweiligen Klinik bzw. alternativ Gründe, weshalb Patienten nicht mit EKT behandelt werden können
- Grundsätzliche Bereitschaft, Patienten auf freiwilliger Basis bzw. gegen den natürlichen Willen mit EKT zu behandeln
- Anzahl der 2018 mit EKT behandelten Patienten an der Klinik inklusive Einschätzung der Wirksamkeit nach Clinical Global Impression Scale – Global Improvement (CGI‑I; [7]), Anzahl der untergebrachten Patienten und Verteilung der Diagnosen an der jeweiligen Klinik
- Allgemeine Einschätzung des Bedarfs an EKT im Maßregelvollzug auf einer visuellen Analogskala (0 = nicht benötigt/verzichtbar, 10 = dringend benötigt/unverzichtbar)

Zur Datenanalyse wurde IBM SPSS Statistics 26 (IBM Corp. Armonk, NY, USA) eingesetzt. In der deskriptiven Statistik wurden Häufigkeiten, Mittelwerte und Standardabweichungen bestimmt. Darüber hinaus wurden zur Bestimmung potenzieller Einflussfaktoren auf Anwendungshäufigkeit bzw. Bedarf an EKT Pearson-Korrelationen auf explorativer Basis (ohne Bonferroni-Korrektur) durchgeführt.

## Ergebnisse

Der Fragebogen wurde von 52 der 79 angeschriebenen Kliniken beantwortet, was einer Rücklaufquote von 66 % entspricht. 46 der 52 Kliniken beantworteten die Frage nach der Anzahl der untergebrachten Patienten. In diesen 46 Kliniken sind insgesamt 7554 Patienten untergebracht, eine Unterscheidung zwischen Unterbringung nach § 63 oder § 64 wurde nicht vorgenommen. Die häufigsten Diagnosen waren schizophrene Erkrankungen (2972 Patienten) und Sucht (2766 Patienten). Neun Kliniken gaben an, ausschließlich Suchtpatienten zu behandeln.

### Eingeschätzte Wirksamkeit und Erfahrungen mit EKT

Die Studienteilnehmer wurden gebeten, ihre Einschätzung zur Wirksamkeit der EKT bei depressiven und schizophrenen Störungen anzugeben. Auf einer visuellen Analogskala von 0 bis 10 erreichte die EKT für die Behandlung depressiver Störungen eine geschätzte Wirksamkeit von 7,8 (*N* = 49; SD 1,645), für die Behandlung von schizophrenen Störungen von 6,12 (*N* = 51; SD 2,242).

Etwas mehr als die Hälfte der Befragten gab an, selbst Erfahrungen in der Behandlung depressiv Erkrankter mit EKT zu haben (53,8 %). In Bezug auf schizophrene Erkrankungen erklärten fast zwei Drittel der Befragten (61,5 %), eigene Erfahrungen als EKT-Anwender zu haben. Neben den eigenen Erfahrungen waren Berichte von Kollegen (69,2 % für Depression; 61,5 % für Schizophrenie) sowie Beschreibungen in der Literatur (65,4 % für Depression; 63,5 % für Schizophrenie) häufige Quelle für die Einschätzung der Wirksamkeit der EKT.

### EKT-Indikation

Bei der Frage, für wie viele Patienten eine EKT-Indikation besteht, wurde ein gewichteter durchschnittlicher Wert von 3,4 % (*N* = 46; Minimum 0 %, Maximum 20 %) ermittelt. Dies entspricht bei den erfassten 7554 untergebrachten Patienten einer Zahl von 257 Patienten. Wie bereits oben erwähnt, werden in neun der 46 Kliniken ausschließlich Suchtpatienten behandelt. Hierbei ist zu beachten, dass im Falle einer Suchterkrankung ohne weitere Komorbidität keine Indikation für die EKT besteht. Bezieht man diese Suchtkliniken nicht in die Rechnung mit ein, erhöht sich die geschätzte Indikation auf 3,93 % (*N* = 37; Minimum 0 %, Maximum 20 %) der untergebrachten Patienten.

### Durchführung von EKT an der eigenen Klinik

In 29 von 52 Kliniken (55,8 %) besteht die Möglichkeit der Behandlung mit EKT. Alle entsprechenden Kliniken gaben an, EKT bei einwilligenden Patienten prinzipiell durchführen zu wollen. Gegen den natürlichen Willen des Patienten würden neun der 29 Kliniken (31 % der EKT-durchführenden Kliniken, 17,3 % der antwortenden Kliniken insgesamt) EKT einsetzen.

Die 22 Kliniken, an denen EKT laut eigener Aussage nicht durchgeführt werden kann, gaben bei möglichen Mehrfachnennungen fehlende strukturelle Voraussetzungen mit Abstand als häufigsten Grund an (*N* = 22; 81,8 %), warum eine EKT-Behandlung an der eigenen Klinik nicht stattfinden kann. Hierbei spielte vor allem die fehlende EKT-Ausrüstung bzw. die fehlende Expertise der Behandler eine Rolle. Juristische Gründe (*N* = 22; 27,3 %) und ethische Bedenken (*N* = 22; 22,7 %) wurden weniger häufig angegeben, wobei sich die ethischen Bedenken vor allem auf eine mögliche EKT-Behandlung gegen den natürlich Patientenwillen bezogen.

### EKT-Behandlungen 2018

12 von 51 diese Frage beantwortenden Kliniken gaben an, im Jahr 2018 mindestens einen Patienten mit EKT behandelt zu haben, was einem Anteil von 23,5 % der Kliniken entspricht (Minimum 1 Patient, Maximum 8 Patienten). Die Gesamtzahl der 2018 im MRV mit EKT behandelten Patienten belief sich auf 32, die sämtlich mit ihrer Zustimmung behandelt wurden. Keine Klinik gab an, eine EKT-Behandlung gegen den natürlichen Patientenwillen durchgeführt zu haben. Das klinische Ansprechen wurde mittels CGI‑I [7] bewertet, hier wurde ein durchschnittlicher Wert von 2,32 Punkten erreicht (1 = sehr viel besser, 2 = viel besser, 3 = nur wenig besser, 4 = keine Veränderung, 5–7 = minimal/viel/sehr viel schlechter).

### Geschätzter Bedarf an EKT im Maßregelvollzug

**Figure 1:**
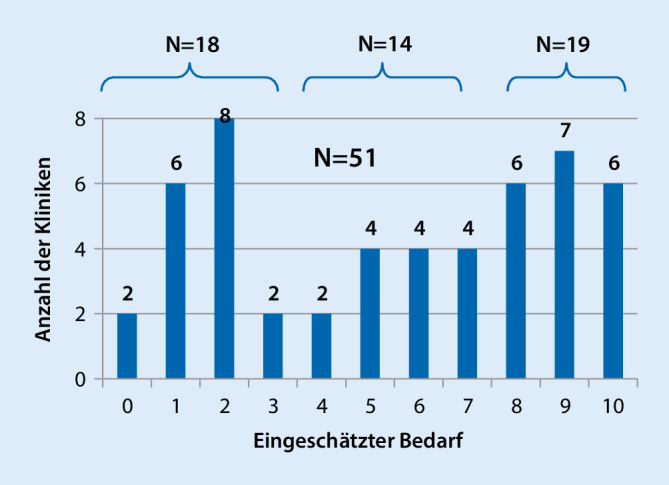

Als abschließende Frage wurden die Leitungen der Kliniken gebeten, mithilfe einer visuellen Analogskala den Bedarf an EKT im MRV anzugeben (0 = nicht benötigt/verzichtbar, 10 = dringend benötigt/unverzichtbar). Es wurde ein Mittelwert von 5,47 (*N* = 51; SD 3,325) erreicht. Ein Ausschluss der neun reinen Suchtkliniken (§ 64) führte dabei zu keiner relevanten Veränderung (*N* = 42; M = 5,48, SD 3,23). Deutlich aussagekräftiger als der Mittelwert ist jedoch die Verteilung (Abb. 1) der Bedarfseinschätzung mit einer deutlichen Abweichung von der Normalverteilung.

### Einflussfaktoren auf die Anwendung der EKT im MRV

Im Rahmen einer explorativen Korrelationsanalyse (unkorrigiert) wurde nach möglichen Einflussfaktoren auf die Durchführungshäufigkeit bzw. den Bedarf an EKT im MRV gesucht. Die Anzahl der tatsächlich in 2018 mit EKT behandelten Patienten korrelierte dabei positiv mit der eingeschätzten Indikationshäufigkeit (r = 0,524, *p* < 0,001). Der angegebene Bedarf an EKT korrelierte positiv mit der Einschätzung zur Wirksamkeit der EKT bei Schizophrenie (r = 0,328, *p* = 0,02), der eingeschätzten Indikationshäufigkeit (r = 0,285, *p* = 0,043) sowie der Anzahl der 2018 tatsächlich mittels EKT behandelten Patienten (r = 0,302, *p* = 0,031).

## Diskussion

Unsere Studie untersuchte erstmalig für Deutschland den gegenwärtigen Stand der Anwendung der EKT im MRV. Zusätzlich wurden die Einschätzungen zum Bedarf an EKT und mögliche Hürden der Etablierung der EKT in der forensischen Psychiatrie erfragt.

Durch den relativ hohen Rücklauf von etwa zwei Drittel der angeschriebenen Einrichtungen sollte der zugrunde liegende Datensatz eine hinreichend repräsentative Einschätzung der aktuellen Anwendung und des Bedarfs an EKT im MRV in Deutschland ermöglichen. Für das Jahr 2018 gaben etwas mehr als die Hälfte der Kliniken an, Patienten prinzipiell mit EKT behandeln zu können. In etwa 41 % dieser Kliniken und in 23 % der antwortenden Kliniken insgesamt wurde auch tatsächlich mindestens ein Patient mit EKT behandelt. Diese Behandlungen führten durchschnittlich zu einer deutlichen Besserung des klinischen Gesamteindrucks der Patienten. Gegen den natürlichen Willen wurden im MRV 2018 keine EKT-Behandlungen durchgeführt, knapp ein Drittel der Kliniken mit verfügbarer EKT gab jedoch an, die Therapie bei Vorliegen der entsprechenden Voraussetzungen grundsätzlich auch gegen den natürlichen Willen anwenden zu wollen. Eine Indikation zur EKT wurde bei insgesamt etwa 4 % der Patienten des MRV gesehen, was hochgerechnet einer Gesamtanzahl von 256 Patienten des MRV entspricht. Die tatsächliche Anwendung der EKT ist somit relativ zu der gestellten Indikation um den Faktor 8 niedriger. Gründe hierfür bestehen offenbar hauptsächlich in strukturell-organisatorischen Aspekten, in geringerem Maße auch in formalen/juristischen Hürden sowie vereinzelt auch ethischen Bedenken.

In 23 % der an unserer Fragebogenstudie beteiligten Kliniken des MRV wurde im Jahr 2018 zumindest ein Patient mit EKT behandelt. Verglichen mit den Kliniken der Allgemeinpsychiatrie (43 %) ist der Wert deutlich niedriger [15]. Bemerkenswert ist außerdem, dass eine relevante Anzahl (21 %) der forensischen Kliniken eine Indikation zur EKT bei ihren Patienten sieht, die Behandlung aber trotz der prinzipiellen Möglichkeit nicht durchführt. In Übereinstimmung hierzu ist die Anzahl der mit EKT behandelten Patienten (32 Patienten im Jahr 2018) verglichen mit der gestellten Indikation (256 Patienten) um den Faktor 8 niedriger.

Hauptgrund für den seltenen Einsatz der EKT im MRV scheinen in erster Linie fehlende strukturelle Voraussetzungen zu sein (fehlendes EKT-Gerät, Anästhesie, Räumlichkeiten, fehlende Erfahrungen und personelle Expertise). Hier könnten Kooperationen mit EKT-Kliniken der Allgemeinpsychiatrie und ggf. anästhesiologischen Praxen ein Ansatz sein. Im fachlichen Austausch mit Kollegen ohne eigene EKT-Erfahrung fällt zudem auf, dass der Raum- und Ausstattungsbedarf für die EKT (Behandlungs- und Aufwachraum) tendenziell eher überschätzt wird. Auch hier könnte die Zusammenarbeit im Sinne eines Peer-Reviews möglicherweise beitragen, Hürden für die Behandlung abzubauen.

Möglicherweise stellen auch die erhöhten Sicherungs- und Betreuungsvoraussetzungen Hindernisse für die Behandlung dar. Häufig werden die EKT-Behandlungen nicht in der forensischen Klinik selbst durchgeführt. Dementsprechend müssen die Patienten aus (hoch-)gesicherten Abteilungen in Kliniken gebracht werden, an denen die EKT durchgeführt werden kann. Auf dem Weg und vor Ort müssen Betreuung und auch Sicherung der Patienten gewährleistet werden. Die Nachbetreuung muss dann wieder auf der gesicherten forensischen Station umgesetzt werden. Dieser Ablauf ist personalintensiv, fordert auch die Sicherung betreffend organisatorische Regelung und setzt die Bereitschaft und vorhandene Ressourcen für die Nachbetreuung voraus.

Ethische Bedenken (22,7 %) und juristische Hürden (27,3 %) wurden zwar deutlich seltener als Hinderungsgründe für die Durchführung der EKT in der forensischen Psychiatrie genannt, dennoch spielen sie vermutlich in dem komplexen Gesamtgefüge des MRV eine relevante Rolle, zumal entsprechende Diskussionen auch in der Allgemeinpsychiatrie geführt werden [24, 26].

Studien haben jedoch zeigen können, dass eine positive Einstellung zur EKT berufsgruppenübergreifend u. a. davon abhängig ist, inwieweit die Befragten Kontakt zu mittels EKT behandelten Patienten hatten [21]. Hierzu passend zeigen unsere Daten eine signifikante Korrelation zwischen den tatsächlich durchgeführten Behandlungen und dem geschätzten Bedarf an EKT in der forensischen Psychiatrie. Die eigene Anwendungserfahrung mit EKT, mitsamt der wie oben beschriebenen durchschnittlich guten Wirksamkeit bezogen auf den klinischen Gesamteindruck, ist somit ein relevanter Faktor, der mit einer höheren Bedarfseinschätzung an EKT einhergeht. Die Erfahrung erfolgreicher EKT-Behandlungen führt nachvollziehbar dazu, die EKT häufiger auch bei anderen Patienten in Erwägung zu ziehen.

Im spezifischen Setting des MRV kommt der Frage nach der Einwilligung in eine medizinische Maßnahme eine besondere Bedeutung zu. Ethisch und juristisch unstrittig dürfte dabei die Zustimmung (oder Ablehnung) des einwilligungsfähigen Patienten sein, ebenso die Einwilligung des rechtlichen Vertreters bei fehlender Ablehnung des nicht einwilligungsfähigen Patienten. Nach dem Bundesverfassungsgerichtsbeschluss zur medikamentösen Zwangsbehandlung in 2011 [6], sind die Voraussetzungen und Bestimmungen in den einzelnen Bundesländern neu geregelt worden. Auf der gesetzlichen Grundlage des Nds. MVollzG ist wie oben beschrieben unter eng definierten Voraussetzungen auch eine Behandlung gegen den natürlichen Willen möglich. Auffällig in diesem Kontext ist der aktuelle Befund, dass im spezifischen Setting des MRV im Gegensatz zur Allgemeinpsychiatrie keine EKT-Behandlung gegen den natürlichen Willen durchgeführt wurde. Ferner erklärte nur etwa jede dritte forensische Klinik, an der EKT durchgeführt werden könnte, prinzipiell auch Patienten gegen ihren natürlichen Willen mit EKT zu behandeln. Aus der Allgemeinpsychiatrie liegt mittlerweile eine relevante Literatur zu Behandlungen einwilligungsunfähiger Patienten gegen den natürlichen Willen vor [2, 3, 11, 17, 22]. In Abwesenheit randomisiert-kontrollierter Studien, die es aus naheliegenden Gründen bei dieser Patientenpopulation nicht geben kann, sprechen diese Fallserien und Fall-Kontroll-Studien konsistent dafür, dass sich die Ansprechraten von durchschnittlich etwa 80 % zwischen freiwillig und (initial) unfreiwillig behandelten Patienten nicht unterscheiden. Ebenso zeigen sich keine Gruppenunterschiede hinsichtlich der nach der Behandlung erhobenen Einstellungen und Zustimmungsraten zur EKT. Die EKT scheint somit auch bei einer schwer und zum Teil chronisch erkrankten Klientel ein wirksames und sicheres Therapieverfahren zu sein, das in der Mehrzahl der Fälle im wiederhergestellten Zustand der Einwilligungsfähigkeit auf retrospektive Zustimmung der betroffenen Patienten stößt.

Diese Datenlage weitgehend außer Acht lassend hat der Bundesgerichtshof (BGH) in einem Beschluss vom 15. Januar 2020 [XII ZB 381/19] festgestellt, dass im konkret verhandelten Fall eines Patienten mit chronifizierter paranoider Schizophrenie die Einwilligung des Betreuers in die zwangsweise Durchführung der Elektrokonvulsionstherapie (EKT) nicht genehmigungsfähig ist [5]. Begründet wurde dies zusammenfassend damit, dass für die gegebene Indikation der chronifizierten (im Gegensatz zur akut exazerbierten oder katatonen) Schizophrenie kein ausreichend breiter „medizinisch-wissenschaftlicher Konsens“ bestehe. Unabhängig davon, dass der Beschluss unter anderem insofern zu kritisieren ist, als er für die Indikationsstellung die 17 Jahre alte Stellungnahme des Wissenschaftlichen Beirats der Bundesärztekammer [12] heranzieht, enthält er jedoch die Feststellung, dass die „Durchführung einer EKT gegen den Widerstand des Patienten“ in anderen Fällen durchaus „kunstgerecht sein kann“. Insofern besteht auch weiterhin die prinzipielle Option der Durchführung der EKT gegen den natürlichen Willen, die, wie bei jedem Grundrechtseingriff erforderlich, im individuellen Fall zu prüfen ist.

National wie international gibt es kaum systematische Literatur zur Anwendung der EKT im MRV. Eine Arbeit aus Dänemark beschreibt *n* = 8 Patienten mit pharmakotherapieresistenter Schizophrenie, die in einer forensischen Einrichtung in einem retrospektiven Zeitraum von sechs Jahren mit EKT behandelt wurden [13]. Bei sechs der acht Patienten bestand eine clozapinresistente Symptomatik, die mittlere Krankheitsdauer war 16 Jahre, die mittlere Dauer der aktuellen Episode 34 Monate. Die psychotische Symptomatik war in allen Fällen von schwerwiegendem aggressivem Verhalten begleitet. Die Patienten erhielten zwischen zwei und 29 Einzelbehandlungen mit EKT. Sechs Patienten sprachen gemäß modifizierter Clinical Global Impression Scale [7] sehr gut auf die Behandlung an, ein Patient gut, bei einem zeigte sich keine Verbesserung. Die Autoren schlussfolgern, dass die EKT bei vorliegenden Positivsymptomen in Kombination mit aggressivem Verhalten eine effektive und sichere Therapie darstellt, und zwar unabhängig von der vorherigen Krankheits- und Episodendauer. In manchen Fällen ermöglichte die Therapie einen Übergang vom stark gesicherten forensischen Setting in die Gesellschaft.

Vor allem vor dem Hintergrund dieses zuletzt genannten Punkts stellt sich die Frage, inwieweit ein weitgehender Verzicht auf EKT im MRV ethisch vertretbar ist, wenn eine evidenzbasierte und leitlinienkonforme Therapieform wie oben beschrieben auch im Setting des MRV einen Beitrag leisten kann, das Therapieziel der bestmöglichen Behandlung und dadurch der Vorbereitung auf ein Leben in Freiheit zu erreichen [19]. Trotz bestehender praktischer, juristischer und zumindest gefühlt auch ethischer Hürden wäre es im Sinne einer evidenzbasierten, zielorientierten, eine Resozialisierung ermöglichenden Behandlung und damit auch im Patientensinne wünschenswert, wenn für die Einrichtungen des MRV ein besserer Zugang zur EKT etabliert werden kann. Die Erfahrungen aus dem Bereich der Allgemeinpsychiatrie zeigen, dass es letztlich der direkte Kontakt zu mit EKT behandelten Patienten bzw. die eigene Behandlung mit EKT ist, der bzw. die sowohl beim Personal als auch bei den Patienten selber zu einer positiven Haltung zur EKT führt [1, 21]. Diese Erfahrungen wären aus den oben genannten Gründen auch den Patienten (und Behandlern) des MRV zu wünschen.

## Limitationen

Unsere Studie diente der Erhebung erster empirischer Daten zur Anwendung der EKT im MRV in Deutschland. Mit dem Ziel eines möglichst hohen Rücklaufs an Fragebögen wurde dieser bewusst kurz gehalten, was in der Konsequenz zu gewissen Limitationen führt. So kann unsere Studie keine Aussagen darüber treffen, welche Patienten mit welchen Diagnosen und welchen möglicherweise prognoserelevanten klinischen sowie personenbezogenen Faktoren (z. B. Alter, spezifische Symptomatik, Grad der Therapieresistenz) behandelt wurden. Auch konkrete Aspekte der EKT-Durchführung (z. B. Anzahl und Frequenz der Behandlungen, Elektrodenposition, Stimulationsdosis, Qualitätsparameter), die jedoch entscheidend für die Wirksamkeit (und Verträglichkeit) der Behandlung sein können, wurden nicht erhoben. Diese Daten sollten zukünftig im Sinne von Fallserien oder besser noch Fall-Kontroll-Studien erhoben werden, um konkrete Effekte der EKT im MRV abbilden zu können. Durch die länderspezifischen gesetzlichen Grundlagen der Unterbringung und Behandlung im MRV ist zudem davon auszugehen, dass etwaige Hürden im Zugang zur EKT und der Genehmigung der EKT bei Patienten im MRV sich ebenso länderspezifisch unterscheiden können. Auch dies könnte spezifisch erhoben und vor ethischem und juristischem Hintergrund im Hinblick auf eine mögliche Ungleichbehandlung von Patienten geprüft werden.

## Zusammenfassung und Fazit für die Praxis

Zusammengefasst besteht hinsichtlich der Elektrokonvulsionstherapie (EKT) als evidenzbasierte und leitlinien-empfohlene Therapie eine deutliche Unterversorgung im Maßregelvollzug (MRV). Zwar kommt die EKT in einigen Einrichtungen des MRV in Einzelfällen zur Anwendung, in den meisten Kliniken scheinen jedoch insbesondere strukturell-organisatorische Aspekte einer indikationsgerechten Anwendung im Wege zu stehen. Diese Nichtverfügbarkeit der EKT als evidenzbasierte und leitlinienkonforme Therapie für sowohl affektive als auch psychotische Störungen ist aus vielfacher Perspektive problematisch. Klinisch stellt die EKT gemäß Literatur sowie unserer eigenen Erfahrung nach bei anderweitiger Therapieresistenz oft das einzige therapeutische Mittel dar, um eine relevante Symptom- und dadurch letztlich auch Prognoseverbesserung bei Patienten auch mit chronifizierten Krankheitsverläufen zu erreichen, was auch für Behandlungen gegen den natürlichen Willen sowie – auf einer noch sehr limitierten Datengrundlage – offenbar auch für Patienten im MRV gilt [13]. Aus ethischen Gesichtspunkten ist zu hinterfragen, ob durch das spezifische Setting des MRV sowie die spezifische rechtliche Grundlage der Unterbringung und Behandlung eine Ungleichbehandlung der Patienten entsteht, da sie aus strukturellen Gründen oder juristischen Hürden zumindest praktisch von einer der wirksamsten Therapien der psychiatrischen Versorgung abgeschnitten sind.

## References

[1] Aoki Y, Yamaguchi S, Ando S (2016). The experience of electroconvulsive therapy and its impact on associated stigma: a meta-analysis. Int J Soc Psychiatry.

[2] Besse M, Methfessel I, Simon A (2019). Electroconvulsive therapy in incapable patients refusing treatment: prevalence, effectiveness, and associated factors. J ECT.

[3] Besse M, Methfessel I, Wiltfang J (2017). Electroconvulsive therapy in nonconsenting patients. Nervenarzt.

[4] Braga RJ, John M, Schooler NR (2019). Continuation electroconvulsive therapy for patients with clozapine-resistant schizophrenia: a pilot study. J ECT.

[5] Bundesgerichtshof (2020) Beschluss vom 15. Januar 2020 – Az. XII ZB 381/19. https://www.bundesgerichtshof.de/SharedDocs/Pressemitteilungen/DE/2020/2020016.html. Zugegriffen: 21.04.2020

[6] Bundesverfassungsgericht (2011) Beschluss des Zweiten Senats vom 23. März 2011 – 2 BvR 882/09 –, Rn. (1–83). http://www.bverfg.de/e/rs20110323_2bvr088209.html. Zugegriffen: 27.02.2020

[7] Busner J, Targum SD (2007). The clinical global impressions scale: applying a research tool in clinical practice. Psychiatry (Edgmont).

[8] Ceus Consulting (2016). Kerndatensatz im Maßregelvollzug, Auswertungen 2006 und 2016.

[9] Deutsche Gesellschaft für Psychiatrie, Psychotherapie und Nervenheilkunde (2005). S3 Praxisleitlinien in Psychiatrie und Psychotherapie. Band 1 – Behandlungsleitlinie Schizophrenie.

[10] Deutsche Gesellschaft für Psychiatrie, Psychotherapie und Nervenheilkunde (2019). S3-Leitlinie Schizophrenie. Langfassung.

[11] Finnegan M, O’Connor S, McLoughlin DM (2018). Involuntary and voluntary electroconvulsive therapy: a case-control study. Brain Stimul.

[12] Folkerts H, Remschmidt H, Saß H (2003). Bekanntmachungen: Stellungnahme zur Elektrokrampftherapie (EKT) als psychiatrische Behandlungsmaßnahme. Dtsch Arztebl.

[13] Kristensen D, Brandt-Christensen M, Ockelmann HH (2012). The use of electroconvulsive therapy in a cohort of forensic psychiatric patients with schizophrenia. Crim Behav Ment Health.

[14] Lally J, Tully J, Robertson D (2016). Augmentation of clozapine with electroconvulsive therapy in treatment resistant schizophrenia: a systematic review and meta-analysis. Schizophr Res.

[15] Loh N, Nickl-Jockschat T, Sheldrick AJ (2013). Accessibility, standards and challenges of electroconvulsive therapy in Western industrialized countries: a German example. World J Biol Psychiatry.

[16] McCall WV (2015). Electroconvulsive therapy and forensic psychiatry. J ECT.

[17] Methfessel I, Sartorius A, Zilles D (2018). Electroconvulsive therapy against the patients’ will: a case series. World J Biol Psychiatry.

[18] Müller J (2019). Maßregelvollzug auf dem Prüfstand. Dtsch Arztebl.

[19] Müller JL, Saimeh N, Briken P (2017). Standards for treatment in forensic committment according to section sign 63 and section sign 64 of the German criminal code: interdisciplinary task force of the DGPPN. Nervenarzt.

[20] Petrides G, Malur C, Braga RJ (2015). Electroconvulsive therapy augmentation in clozapine-resistant schizophrenia: a prospective, randomized study. Am J Psychiatry.

[21] Scholz-Hehn AD, Müller JC, Deml R (2019). Factors influencing staff’s attitude toward electroconvulsive therapy: a comparison of new versus experienced electroconvulsive therapy clinics. J ECT.

[22] Takamiya A, Sawada K, Mimura M (2019). Attitudes toward electroconvulsive therapy among involuntary and voluntary patients. J ECT.

[23] Ward HB, Szabo ST, Rakesh G (2018). Maintenance ECT in schizophrenia: a systematic review. Psychiatry Res.

[24] Wiesing U, Fallgatter AJ (2018). Rationality and freedom in medicine: the case of electroconvulsive therapy. Nervenarzt.

[25] Witzel J, Held E, Bogerts B (2009). Electroconvulsive therapy in forensic psychiatry—ethical problems in daily practice. J ECT.

[26] Zilles D, Koller M, Methfessel I (2018). Ethics, evidence and electroconvulsive therapy. Nervenarzt.

